# Discrimination of Thermophilic Proteins and Non-thermophilic Proteins Using Feature Dimension Reduction

**DOI:** 10.3389/fbioe.2020.584807

**Published:** 2020-10-22

**Authors:** Zifan Guo, Pingping Wang, Zhendong Liu, Yuming Zhao

**Affiliations:** ^1^School of Aeronautics and Astronautic, Institute of Fundamental and Frontier Sciences, University of Electronic Science and Technology of China, Chengdu, China; ^2^School of Life Science and Technology, Harbin Institute of Technology, Harbin, China; ^3^School of Computer Science and Technology, Shandong Jianzhu University, Jinan, China; ^4^Information and Computer Engineering College, Northeast Forestry University, Harbin, China

**Keywords:** support vector machine, thermophilic proteins, feature dimension reduction, amino acid, feature selection

## Abstract

Thermophilicity is a very important property of proteins, as it sometimes determines denaturation and cell death. Thus, methods for predicting thermophilic proteins and non-thermophilic proteins are of interest and can contribute to the design and engineering of proteins. In this article, we describe the use of feature dimension reduction technology and LIBSVM to identify thermophilic proteins. The highest accuracy obtained by cross-validation was 96.02% with 119 parameters. When using only 16 features, we obtained an accuracy of 93.33%. We discuss the importance of the different characteristics in identification and report a comparison of the performance of support vector machine to that of other methods.

## Introduction

Temperature is a critical condition for life. Proteins are less stable than other macromolecules, and temperature changes can easily lead to protein denaturation, which can lead to cell death (Kumar et al., 2000). Thus, it is important to develop a highly efficient method for predicting protein thermophilicity, which will contribute to the design of stable proteins. The properties of many proteins are related to their thermal stability. Studies have shown that the thermal stability of proteins is influenced by ion number, salt bridge presence, amino acid composition (AAC), dipeptide composition (DPC), and other factors (Sadeghi et al., 2006; Wang H. et al., 2018; Yin et al., 2020). Zhang and Fang (2006), Li et al. (2018), and Wang Y. et al. (2020) found significant differences in the presence of some dipeptides between thermophilic and mesothermal proteins. In addition, Gromiha et al. (1999) found that protein stability was associated with the balance between packing and solubility.

Many studies have been conducted on methods of distinguishing thermophilic proteins from normal-temperature proteins based on protein properties. Liang et al. (2005) proposed an amino acid coupling model with strong statistical ability to distinguish between thermophilic proteins and mesophilic proteins. LogitBoost Classifier and 20 features were used to distinguish thermophilic proteins by Zhang and Fang (2007) which achieved an overall classification accuracy reaching 88.9%. Montanucci et al. (2008) applied support vector machine (SVM) to investigate the impacts of mutations on the thermal stability of proteins, and with jackknife cross-validation, they achieved a prediction accuracy of 88%. Recently, Lin and Chen (2011) used feature selection technique and SVM with 30 parameters to predict thermotropic proteins, and the overall accuracy reached 93.27%. These methods have achieved good accuracy, but there remains room for improvement in the number of features used and prediction performance.

In this work, we used the data set of Lin and Chen (2011) after eliminating redundancy to distinguish between thermophilic proteins and non-thermophilic proteins. After feature extraction, MRMD2.0 was applied for feature selection and dimension reduction, and LIBSVM was used to obtain the optimal parameters of the model and establish the prediction model. Finally, from the results of cross-validation, both the number of features and the prediction accuracy were improved; the overall prediction accuracy with only 16 features in AAC was increased to 93.33%, and the highest overall accuracy, attained with 119 parameters, reached 96.02%. In addition, we analyzed the importance of features and demonstrated the strong performance of SVM by comparing this method with other methods.

## Materials and Methods

### Data Sets

In this article, we conducted prediction experiments using two groups of data, namely, a group of thermophilic protein data and a group of non-thermophilic protein data. The data sets were collected by Lin and Chen (2011). Generally, thermophilic proteins and non-thermophilic proteins derive from the corresponding biosome, and optimum growth temperature is the key feature used to distinguish thermophilic and non-thermophilic proteins. Therefore, we used 60°C as the minimum optimum growth temperature for thermophilic proteins and 30°C as the maximum optimum growth temperature for non-thermophilic proteins to avoid the problem of protein denaturation. As a result, 136 prokaryotic genomes conforming to the standard were selected, and their protein sequences were obtained from the Universal Protein Resource.

Next, we screened the protein sequences to increase the quality of the data sets. The filtering process employed the following criteria: (1) the sequence must have manual annotation and evaluation; (2) the protein sequence cannot include ambiguous residue; (3) the sequences cannot be fragments of other proteins; and (4) the sequence cannot be deduced from prediction or homology. After the above screening process, we obtained a total of 1,250 non-thermophilic proteins and 1,329 thermophilic proteins. Next, highly similar sequences were removed by employing the CD-HIT program, resulting in 793 non-thermophilic proteins and 915 thermophilic proteins.

### Feature Extraction

**Figure 1:**
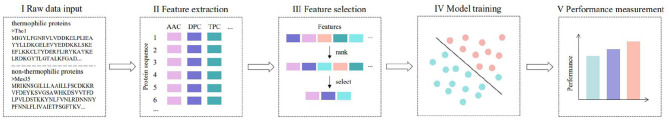
Study flowchart. (I) The original protein sequence is input for feature extraction. (II) A feature extraction algorithm is used to obtain feature descriptors of each protein. (III) MRMD2.0 is used to rank the importance of features and select features. (IV) Support vector machine is used for parameter optimization and training model establishment. (V) Three parameters are used to evaluate the performance of the model: sensitivity (SE), specificity (SP), and accuracy (ACC).

Before protein prediction, the features of the protein sequences were extracted to construct the feature vectors (Figure 1). For this purpose, iFeature was used, which is a utility toolkit based on python to obtain miscellaneous numerical feature representation schemes for protein sequences (Chen et al., 2018). When using iFeature, users can combine various feature clustering, feature selection, and dimension reduction algorithms to promote the analysis of feature importance and model training. iFeature has been widely tested to ensure the validity of our calculations to further ensure the strength of our work.

We used iFeature to extract the features of the protein sequences from our data set, including AAC (Bhasin and Raghava, 2004; Pan et al., 2018; Chen et al., 2019b; Liu et al., 2019; Shen et al., 2019b; Tang et al., 2019; Li Y. H. et al., 2020), C/T/D composition (CTDC), C/T/D transition (CTDT), conjoint triad (CTriad), dipeptide deviation from the expected mean (DDE) (Saravanan and Gautham, 2015), DPC (Saravanan and Gautham, 2015; Chen et al., 2019a), tripeptide composition (TPC), composition of k-spaced amino acid pairs (CKSAAP), grouped dipeptide composition (GDPC), and grouped tripeptide composition (GTPC). The following is a concise explanation of the feature extraction protocol. In all of the following formulas, *n* denotes the length of the protein sequence.

#### AAC

AAC refers to the frequency of each amino acid in a protein or peptide sequence. There are 20 kinds of naturally occurring amino acids, namely, ACDEFGHIKLMNPQRSTVWY, and their frequencies in a sequence can be calculated by the following formula:

$$\begin{array}{l} f\left(i\right)=\frac{n\left(i\right)}{n},\;i\in \left\{A,C,D,E,F,\ldots ,W,Y\right\} \end{array}$$

where *n*(*i*) refers to the number of occurrences of amino acid *i*.

#### DPC

DPC refers to the frequency of dipeptide combinations in a protein or peptide sequence, which yields 400 descriptors (Cheng J. H. et al., 2018; Tang et al., 2018). It is defined by the following formula:

$$\begin{array}{l} \;f\left(x,y\right)=\frac{n_{xy}}{n-1},\;x,y\in \left\{A,C,D,E,F,\ldots ,W,Y\right\} \end{array}$$

where *n*_*xy*_ refers to the number of dipeptides denoted by amino acids *x* and *y*.

#### TPC

TPC refers to the frequency of tripeptide combinations in a protein or peptide sequence, which yields 8,000 descriptors (Tan et al., 2019; Zhu et al., 2019). It is defined by the following formula:

$$\begin{array}{l} f\left(x,y,z\right)=\frac{n_{xyz}}{n-2},\;x,y,z\in \left\{A,C,D,E,F,\ldots ,W,Y\right\} \end{array}$$

where *n*_*xyz*_ refers to the number of tripeptides denoted by amino acid combination *x*, *y*, and *z*.

#### DDE

The DDE eigenvector is constructed by calculating three parameters: dipeptide composition (*D*_*c*_), theoretical mean value (*T*_*m*_), and theoretical variance (*T*_*v*_). These three parameters and DDE are calculated as follows:

$$\begin{array}{l} D_{c}\left(x,y\right)=\frac{n_{xy}}{n-1},\;x,y\in \left\{A,C,D,E,F,\ldots ,W,Y\right\} \end{array}$$

where *n*_*xy*_ refers to the number of dipeptides displayed by amino acid combination *x* and *y*.

$$\begin{array}{l} T_{m}\left(x,y\right)=\frac{C_{x}}{C_{n}}\times \frac{C_{y}}{C_{n}},\;x,y\in \left\{A,C,D,E,F,\ldots ,W,Y\right\} \end{array}$$

where *C*_*x*_ and *C*_*y*_ are the number of codons encoding the first and second amino acids, respectively, in dipeptide “*x, y*,” and *C*_*n*_ is the total number of possible codons remaining after removing the 3 terminated codons.

$$\begin{array}{l} \;T_{v}\left(x,y\right)=\frac{T_{m}\left(x,y\right)\left(1-T_{m}\left(x,y\right)\right)}{n-1},\;\\ \;x,y\in \left\{A,C,D,E,F,\ldots ,W,Y\right\}\\ DDE\left(x,y\right)=\frac{D_{c}\left(x,y\right)-T_{m}\left(x,y\right)}{\sqrt{T_{v}\left(x,y\right)}} \end{array}$$

#### GDPC

The GDPC encoding is a change of the DPC descriptor that includes a total of 25 descriptors, defined as follows:

$$\begin{array}{l} f\left(x,y\right)=\frac{n_{xy}}{n-1},\;x,y\in \left\{g1,g2,g3,g4,g5\right\} \end{array}$$

where *n*_*xy*_ refers to the number of dipeptides denoted by amino acid groups *x* and *y*.

#### GTPC

The GTPC is another change of TPC descriptor, which consists of a total of 125 descriptors and is defined as follows:

$$\begin{array}{l} f\left(x,y,z\right)=\frac{n_{xyz}}{n-2},\;x,y,z\in \left\{g1,g2,g3,g4,g5\right\} \end{array}$$

where *n*_*xyz*_ refers to the number of tripeptides denoted by amino acid combination *x*, *y*, and *z*.

#### CTD

CTD features represent the structural or physicochemical distribution patterns of amino acids in protein or peptide sequences (Dubchak et al., 1999; Tang et al., 2020). Thirteen types of physicochemical properties were used to calculate these characteristics, including hydrophobicity, standardized van der Waals volume, solvent accessibility, polarity, secondary structure, polarizability, and charge. These descriptors were computed by the following procedures: (1) the amino acid sequences were changed into residues with certain structural or physicochemical properties; (2) according to the main cluster of Tomii and Kanehisa (1996) amino acid index, the 20 amino acids were divided into 3 groups according to 7 physicochemical properties.

##### CTDC

After all 20 amino acids are divided into three groups, the composition descriptor is composed of 3 values, which are the total percentages of group 1, group 2, and group 3 of the protein sequences. The descriptor is calculated as follows:

$$\begin{array}{l} C\left(x\right)=\frac{n\left(x\right)}{n},\;x\in \left\{\text{group }1,\text{group }2,\text{group }3\right\}\; \end{array}$$

where *n*(*x*) refers to the number of occurrences of amino acid *x* in the encoded sequence.

##### CTDT

The transformation descriptor T also contains three values. The transition from group 1 to group 2 is the percentage frequency of a residue from group 1 followed by a residue from group 2 or a residue from group 2 followed by a residue from group 1. Transformations between group 2 and group 3 and between group 3 and group 1 are defined in a similar manner. The transformation descriptor can be calculated as follows:

$$\begin{array}{l} \;T\left(x,y\right)=\frac{n\left(x,y\right)+n\left(y,x\right)}{n-1},\;\\ \;x,y\in \left\{\left(\text{group }1,\text{group }2\right),\left(\text{group }2,\text{group }3\right),\left(\text{group }3,\text{group }1\right)\right\} \end{array}$$

where *n*(*x, y*) and *n*(*y, x*) refer to the numbers of dipeptides denoted by “*x, y*” and “*y, x*,” respectively, in the protein sequence.

### Feature Selection

Feature selection is an important step in the process of protein classification (Figure 1) (Feng et al., 2017; Cheng, 2019; Liu, 2019; Yang W. et al., 2019; Zheng et al., 2019; Wang M. et al., 2020; Yang et al., 2020b; Zhao et al., 2020). MRMD2.0 is a very deep feature selection method, which uses the concept of the PageRank algorithm and is combined with methods such as analysis of variance (Scheffe, 1960), minimal redundancy and maximal relevance (Ding and Peng, 2005), maximal information coefficient, and least absolute shrinkage and selection operator (Xu et al., 2017). As a result, MRMD2.0 integrates seven different feature ranking algorithms with PageRank algorithm and detects optimized dimensionality with forward adding strategy. PageRank algorithm was originally used to attach weight value to each target page: pages with large weight values are displayed in the front, whereas pages with small weight values are displayed in the back. Similarly, MRMD2.0 uses PageRank algorithm and several other feature ranking algorithms to generate a corresponding weight value for each feature to form a ranking of the importance of all features.

In this study, MRMD2.0 was used to select features and reduce the dimension of the obtained features to improve the feature prediction ability. By treating each group of features in the previous step with MRMD2.0, we obtained the combination of features with the highest classification accuracy and the importance ranking of each group of features. Generally, the combination of features with the highest classification accuracy has fewer dimensions, so we refer to this process as feature dimension reduction. Based on the classification performance, we ranked the group of features. After combining the features with good classification performance, we applied MRMD2.0 to select them again. Finally, after comparing the results, we obtained the combination of features with the best classification ability.

In addition, we applied MRMD2.0 to obtain the importance ranking of features. On the rank list, higher-ranked features are more predictive; accordingly, we identified the most important features for the classification of thermophilic proteins and non-thermophilic proteins. The resulting information enhances our knowledge of the properties of proteins and can aid the construction of stable proteins in protein engineering.

### LIBSVM

In this study, LIBSVM was used to construct models and make predictions (Figure 1). LIBSVM is an effective SVM pattern recognition and regression software package designed by Chih-Jen Lin, a professor at Taiwan University, and has been applied in many fields (Lin et al., 2012; Liu et al., 2012, 2017; Ding et al., 2017; Zeng et al., 2017; Wei et al., 2018, 2019; Xu et al., 2018b,c; Cheng et al., 2019b; Deng et al., 2019; Liang et al., 2019; Shen et al., 2019b,a; Su et al., 2019; Yang H. et al., 2019; Li F. et al., 2020; Wang H. et al., 2020; Yang et al., 2020a; Zhang et al., 2020). Before training SVM on a problem, the parameters must be specified (Jiang et al., 2013; Zhao et al., 2015, 2017). We selected the best parameters, C and g, through a simple tool provided by LIBSVM for evaluating a grid of parameters. The accuracy for each parameter setting is obtained in LIBSVM, allowing the parameters with the highest cross-validation accuracy to be determined. Next, we trained the whole data set with the best parameters C and g to obtain the prediction model. Finally, we tested and predicted our data set with the obtained model.

### Performance Measurement

We used three commonly used indicators to evaluate model performance: sensitivity (SE), specificity (SP), and accuracy (ACC) (Figure 1) (Wang et al., 2010; Wei et al., 2017a,b; Zhang et al., 2018; Cheng et al., 2019a; Ding et al., 2019a; Junwei et al., 2019; Liang et al., 2019; Liu and Li, 2019; Tian et al., 2019; Jia et al., 2020; Liu and Chen, 2020; Li J. et al., 2020; Lv et al., 2020; Wang Z. et al., 2020). They are described as follows:

$$\begin{array}{l} \text{ SE}=\frac{\text{TP}}{\text{TP}+\text{FN}}\\ \text{ SE}=\frac{\text{TP}}{\text{TP}+\text{FN}}\\ \text{ACC}=\frac{\text{TP}+\text{TN}}{\text{TP}+\text{FN}+\text{TN}+\text{FP}} \end{array}$$

where TN, TP, FN, and FP refer to the numbers of correctly predicted non-thermophilic proteins, correctly predicted non-thermophilic proteins, incorrectly predicted non-thermophilic proteins, and incorrectly predicted thermophilic proteins, respectively. SE and SP indicators measure the predictive ability of a model in positive and negative situations, respectively, and ACC is used to evaluate the overall performance of a prediction model (Wang et al., 2008; Zou et al., 2017a,b; Cheng L. et al., 2018; Wang G. et al., 2018; Xue et al., 2018; Xu et al., 2018a, 2019; Ding et al., 2019b; Shen et al., 2019b; Yang, 2019; Zeng et al., 2019; Fu et al., 2020; Hong et al., 2020).

## Results and Discussion

### Identification of Protein Thermostability

The results of feature selection by using MRMD2.0 are shown in Table 1. Among them, features with good classification performance include AAC, DPC, CTDC, and dipeptide deviation from the expected mean. However, although the classification ACC of dipeptide deviation from the expected mean after dimension reduction reached 85.6%, it had 365-dimensional features. Considering the excessive dimension and the unexceptional performance, only AAC, DPC, and CTDC were subsequently combined for classification.

**Table 1:** The results of feature selection by using MRMD2.0.

**Feature**	**Dimensions**	**Accuracy (%)**
AAC	16/20	87.94
DPC	103/400	87.00
DDE	365/400	85.60
CTDC	33/39	85.01
CTDT	39/39	80.50
CTriad	338/343	79.80
CKSAAP	143/150	79.04
GTPC	107/125	78.63
GDPC	13/25	78.57
TPC	1,008/1023	77.11

*The two numbers in the second column of the table are the number after dimension reduction and the number before dimension reduction*.

Next, based on LIBSVM and grid parameter optimization, we used various combinations of these three features to construct models and perform cross-validation for our data sets. The results are shown in Table 2. The overall ACC of three schemes is higher than that of Lin and Chen (2011) (93%).

**Table 2:** The results of classification using SVM and various feature combinations.

**Feature combination**	**SE (%)**	**SN (%)**	**Accuracy (%)**
The method of Lin and Chen (2011)	93.77	92.69	93.27
AAC (16)	93.44	93.19	93.33
AAC (16) + CTDC (33)	93.77	92.81	93.33
AAC (16) + DPC (103)	95.85	96.22	96.02

*The numbers in parentheses in the first column of the table represent the number of arguments to the feature preceding the parentheses*.

Initially, we used AAC with 16 dimensions alone to build a prediction model for the data set, achieving an overall ACC rate of 93.33% through cross-validation, which is slightly higher than that of Lin and Chen (2011). In addition, Zhang and Fang (2006) and Gromiha and Suresh (2010) used all 20 amino acids composition to predict the thermostability of protein, and their overall ACC was 90.5 and 89%, respectively. Furthermore, Wang and Li (2014) enhanced the ACC to 95% by selecting 9 AAC and 38 DPC using a genetic algorithm. In contrast, the scheme used only 16 parameters, but the ACC reached 93.33%, which is fewer than the dimensions used in previous studies. The results show that AAC plays an important role in the identification of thermophilic proteins.

The top two features in Table 3 were AAC and DPC. The model constructed with 16 parameters of AAC and 103 parameters of DPC achieved the highest overall ACC of 96.02%. The SE and SP of this method were 95.85 and 96.22%, respectively, which indicates that the predictive ability of this model in both positive and negative situations is excellent.

**Table 3:** The results of classification accuracy using LIBSVM and various combinations of important features.

**Dimension**	**Feature**	**Accuracy (%)**
1	K	76.41
2	K + D	77.50
3	K + D + LK	78.29

*A plus sign in the second column of the table indicates the use of these characteristics for model training and classification. For example, “K + D” indicates the modeling and classification of the data sets with the two-dimension characteristics K and D*.

In addition, we used the combination of AAC with 16 dimensions and CTDC with 33 dimensions to build a prediction model and obtained the same overall ACC as the first model. However, this second model had higher SE and lower SP than the first model, indicating that it was slightly inferior to the model built with 16 dimensions of AAC.

### Feature Importance

We aimed to identify the most important features of the method with 119 parameters that can achieve the highest ACC and analyze them. To assess feature importance, first, we used MRMD2.0 to rank all 119 features by importance. We found that the top three features were K, D, and LK (Feature K is the percentage of lysine in the amino acid sequence, feature D is the percentage of aspartic acid in the amino acid sequence, and feature LK is the percentage content of the dipeptide consisting of leucine and lysine in the amino acid sequence). These three features are arguably the most predictive among the 119 features for the classification of thermophilic proteins.

Next, to obtain the classification performance of the above features, we used one-dimensional (K), two-dimensional (K and D), and three-dimensional (K, D, and LK) features to classify our data set based on LIBSVM. The results are shown in Table 3.

**Figure 2:**
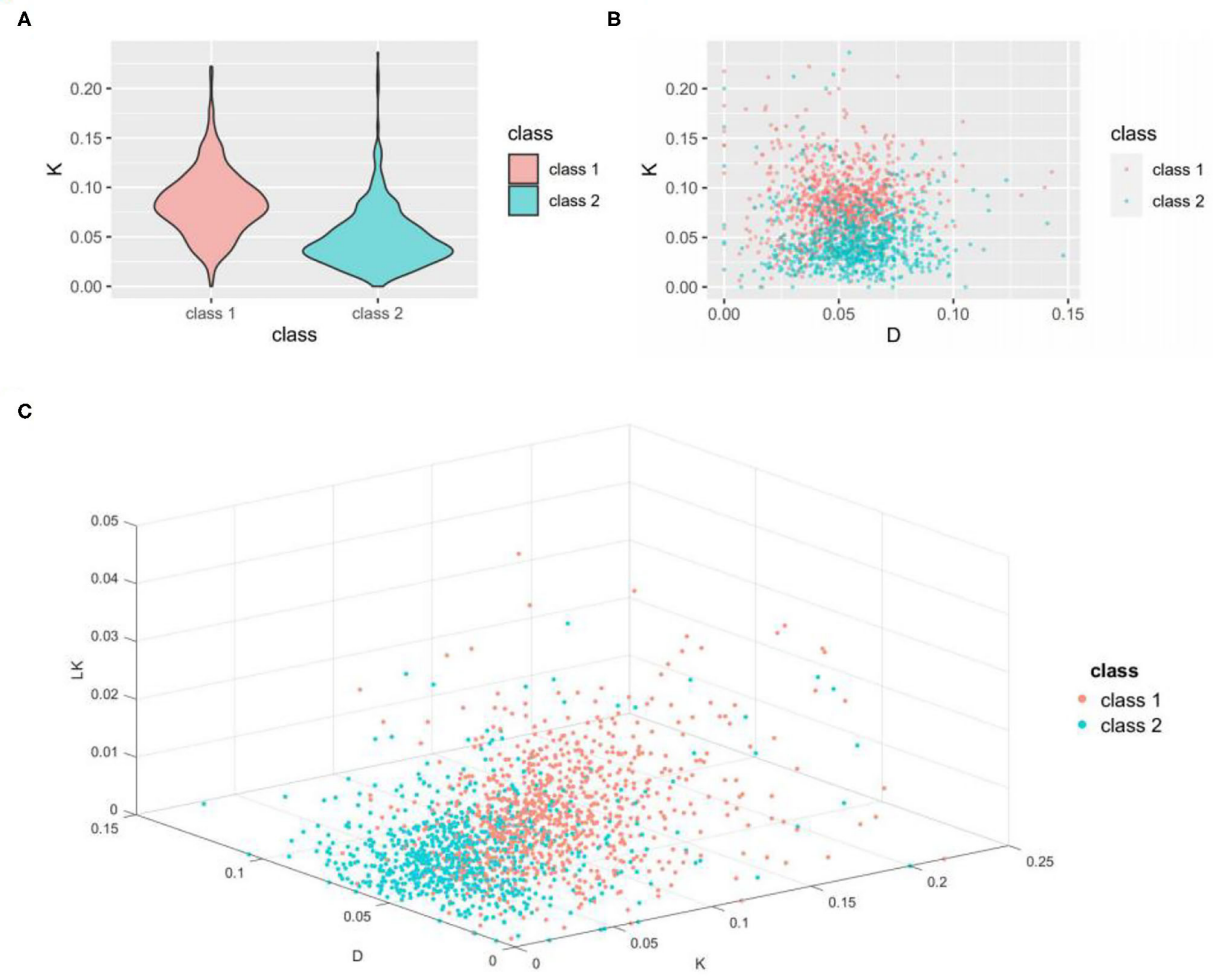
Visualization of the ability of important features to classify thermophilic and non-thermophilic proteins. **(A)** is a violin diagram of the K feature. **(B)** is a scatter diagram of the K feature and D feature. **(C)** is a 3D scatter diagram of the K, D, and LK features. K is the percentage of lysine in the amino acid sequence, D is the percentage of aspartic acid in the amino acid sequence, and LK is the percentage content of the dipeptide consisting of leucine and lysine in the amino acid sequence.

As seen from Table 3, the classification ACC of the K feature alone reached 76.41%, whereas the ACC achieved with K combined with D and LK was only slightly greater. To better analyze the classification ability of these three important features, we constructed a violin diagram, scatter diagram, and 3D scatter diagram for the 1-, 2-, and 3-dimension features. The results are shown in Figure 2.

As seen from Figure 2A, the K value of the thermophilic proteome is concentrated ~0.08, whereas the K value of the non-thermophilic proteome is concentrated ~0.03. These results indicate that the K feature can well distinguish thermophilic proteins from non-thermophilic proteins, a finding of great significance for the identification of the thermophilic properties of proteins. All three panels reveal obvious differences in the distribution pattern between the two data sets, which indicates that these features have strong recognition ability and good performance in distinguishing thermophilic proteins from non-thermophilic proteins, as shown in Table 3.

### Comparison With Other Classification Methods

To reveal the advantage of our method, we applied six other classification methods to train our data sets based on the Waikato environment for knowledge analysis (Weka) tool (Witten and Frank, 2002): logistic, random forest, BayesNet, logistic model trees (LMTs), J48, and reduced error pruning tree (REPTree).

We used the combination with the highest overall ACC in this article (16 features in AAC and 103 features in DPC) as the input, and we used the above classifiers to predict the data set to obtain the SE, SP, and ACC of each method. To ensure a robust comparison, we also used cross-validation to predict the data set. By comparing the performance of different methods, the performance of different classifiers was evaluated. The prediction results of each method applied to the data set are shown in Table 4.

**Table 4:** The performance of different classification methods in the prediction of the data sets.

**Classification method**	**SE (%)**	**SN (%)**	**Accuracy (%)**
SVM (this article)	95.85	96.22	96.02
LMT	92.35	90.29	91.40
Logistic	91.15	88.90	90.11
Random Forest	91.69	87.51	89.75
BayesNet	88.08	86.25	87.24
REPTree	83.60	84.62	84.07
J48	83.50	80.33	82.03

**Figure 3:**
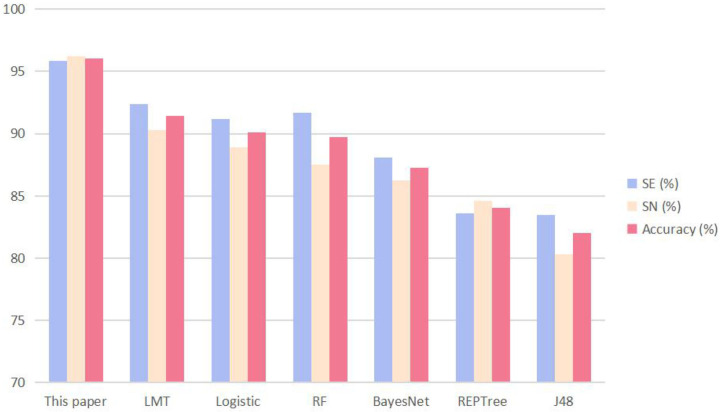
The performance of the method described in this article and other six predictors when the input is 16 parameters of amino acid composition and 103 parameters of dipeptide composition. The performance metrics are sensitivity (SE), specificity (SP), and accuracy (ACC).

It can be seen from Table 4 that the SVM we used in this study achieved the best performance; the SE, SP, and ACC of the other methods were all lower than those of the SVM method of this article. To visualize the data, we constructed a cluster histogram of the performance of the different methods, shown in Figure 3.

The advantage of using SVM to predict data sets is apparent from the histogram.

## Conclusion

In this article, we distinguished 915 thermophilic proteins and 793 non-thermophilic proteins. We applied iFeature to extract the features of the protein sequences. MRMD2.0 was used to reduce the dimensions of features and select the ones that performed the best. LIBSVM was used to optimize the parameters and establish the prediction model. As a result, the overall ACC was improved, which reached 96.02% under cross-validation. Furthermore, we constructed a prediction model by LIBSVM with 16 parameters, and the ACC determined by cross-validation was 93.33%. In addition, we found that the K feature played a significant role in the identification. Finally, we demonstrated the advantage of SVM by comparing its performance with that of other methods. We aim to analyze information, such as the family of misclassified proteins, to optimize our method in the future.

## Data Availability Statement

Publicly available datasets were analyzed in this study. This data can be found here: doi: 10.1016/j.mimet.2010.10.013.

## Author Contributions

ZG made the design of the subject and the whole idea of the whole experiment, did comparative experiments, and the analysis of the experiment. PW did experimental data analysis. ZL and YZ analyzed the results of the experiment and made some improvements to this paper. All authors contributed to the article and approved the submitted version.

## Conflict of Interest

The authors declare that the research was conducted in the absence of any commercial or financial relationships that could be construed as a potential conflict of interest.
